# It’s up to Texas

**Published:** 1907-04

**Authors:** 


### Abstracts and Selections

### It's Up to Texas.

In the *California State Medical Journal,* February issue, the editor takes a slap at the independent medical journals of Texas for their policy toward advertisers. The Texas journalists are quite capable of taking their own part, and we shall not presume to enter the quarrel; but we must take exception to the assumption that the restrictions deemed proper for an ideal "organization (?) journal" (there appears to be no standard) are necessarily applicable to independent publications. The editor of the former is independent of his readers (who must take his journal whether they like it or not) as long as he can muster the support that keeps him in office. He is independent of advertising and all other financial matters, since the organization is responsible for the bills. His journal being the public representative of the organization, it is incumbent on him to uphold to the highest their ideals.

The independent journalist represents and is responsible to himself. His standards are his own. If these do not suit his subscribers they quit. If his readers fail to use the articles advertised, "returns" will not pay the bills, the advertiser drops out, and there you are. Let enough do this and the journal is down and out. Its editor and publisher aren't privileged to tell advertisers they must use this journal or get it in the neck; neither may he tell the medical profession, his constituents, they are "skates" if they use anything not advertised in his journal, and "scabs" if they presume to use anything not endorsed by the "Council of Pharmacy and Chemistry." No, he has to "toe the line," to get and keep both the subscriber and the advertiser—must be fair and just to all and do the best he can for himself.

If he believes that certain advertised remedies are not good enough, are not true to name or formula—are not honest (and he should be very careful about this), he will try to educate his readers up to his own standpoint, and then they quit using the objectionable goods. He does not shelter himself behind the coward's bulwark, but comes out into the open and battles to live or die as his prowess may determine. A free man, choosing for himself, leaves to his readers the same privilege. If, knowing he is right, he is unable to convince his readers of that fact, he is not qualified for the position; and this applies with double force to the editors of organization journals as well. Whenever a leader attempts to use force, rather than persuasion or argument, to bring people to his views, he confesses his weakness and incompetency. It is the cry of impotent mediocrity; the weapon of bureaucracy, the abhorrence of the multiplying thousands who believe in the "square deal."

Just now we are interested to see how much Philip Mills Jones will accomplish, in trying to get the medical press of Texas by the ears,* through this unwarranted attack on friends Kibbie and Daniel for the ads they carry and the way they conduct their journals. How easy it is to criticise others; especially so for thusly-inclined Jones with the great State Medical Society of splendid California at his back to pay his bills.

It is true that the *Texas State Journal* republished the stuff, but they kept wisely still about it. Both they and Jones would have to "clean house" materially to stack up to the theory on which the criticism was made.

The attitude to be taken by the Texas profession is awaited with general interest.

Will the other Texas journals stand by their brethren, or will local jealousies divide the profession? Will the readers demand of the local independent journals the same restrictions the (Czar) managers of the organization publications choose to impose? Or will Texas physicians resent the interference and stand on their independence? Possibly, in view of the self-confessed failure of Jones to whip the California physicians into line on this matter, they may suggest that his energies are needed at home.

What's the use of scrapping? Be fair and square, work hard, be honest with yourselves and generous to others, and you'll come out all right. It is what we think and what we do that make us what we are.—*Advance sheets, editorial in May number, American Journal Clinical Medicine.*

We have not seen this "attack," nor have we seen a copy of the California Tentacle since the earthquake. It must have addled Jonesey's brain. No one takes "This-here-Jones" seriously. I regard him as a kind of a *joke*. Simmons and the Self-Righteous Council of Pharmacy doubtless find a use for him, and it goes without saying that he jumps as the strings are pulled. He *has* to, or *lose his job*. DANIEL.

---

*There is but one other independent Medical Journal in Texas, the *Texas Medical News*, Dr. M. M. Smith, Austin, and he is in line with the independents, and carries the same ads that "friends Kibbie and Daniel" do, and is in harmony with them on this subject.—DANIEL.

